# Stress distribution during orthodontic maxillary canine retraction using 3D finite element analysis

**DOI:** 10.6026/973206300210220

**Published:** 2025-02-28

**Authors:** Parul Singh, Chinki Bansal, Gunjan Kaushik, Utkarsh Shrivas, Kuldip Singh, Ramesh Kumar

**Affiliations:** 1 Department of Orthodontics and Dentofacial Orthopaedics, Maharana Pratap College of Dentistry & Research Centre, Gwalior, Madhya Pradesh, India; 2 Private Consultant Orthodontist, US Dental Clinic, Chhatarpur, Madhya Pradesh, India; 3 Dental Officer, C.H.C. Barua Sagar, Jhansi, Uttar Pradesh, India; 4 Private Consultant Orthodontist, SAH Dental Clinic and Orthodontic Centre, Patna, Bihar, India

**Keywords:** Maxillary canine retraction, stress distribution, periodontium, orthodontic

## Abstract

The stress distribution in the periodontium during maxillary canine retraction between the forces applied at canine orthodontic bracket and at power arm using 3D finite element analysis (FEA). 3D FEA for power arm, archwire, orthodontic bracket and periodontium was built independently using the ANSYS software. Maximum stress areas in periodontium was after 150 gm force application at power arm soldered to canine bracket at 13 mm and minimum stress area in periodontium was with force application at canine bracket hook. Maximum principle stress (tension side) and minimum principle stress (compression side) observed in the periodontium at power arm soldered to canine bracket at 9mm and minimum at canine bracket hook.

## Background:

Application of an optimum force on a tooth in orthodontics produces stresses and strains in the periodontium [1, 2-3]. As a result, a doctor needs to comprehend the periodontium's distribution of stresses and application of force mechanism. Tooth movements arise from a biological reaction triggered by pressure in the periodontal ligament, which causes alveolar bone resorption and deposition [4, 5-6]. An 8:1 up to 10:1 moment to force proportion is necessary for the bodily motion of a tooth. Additionally, it was noted that a tooth is said to be translated when all of its points advance in a parallel, linear fashion according to the path of force [7, 8- 9]. The authors noted that according to the interaction between the force's trajectory of action and the tooth's center of resistance (CR), forces imparted to a tooth can cause translation movement, rotation movement, or a combination of translation movement and rotation movement [10, 11- 12]. Many different bracket compositions and strategies have been created and updated since the initial introduction of the Andrews straight wire apparatus [13, 14- 15]. In sliding orthodontic mechanics, ideal retraction pressures can be provided anywhere on the vertical plane by a power limb that propels the teeth in an already programmed trajectory to achieve physiological movement, but in closing looping mechanics, triggered loop pressures would only function at the bracket position [11, 12, 13-14]. Because vertical position of retraction pressures may be easily modified by connecting different lengths of power limbs to an arch wire, sliding orthodontic mechanics provide a chance to optimize the system of forces required for physiological shifting of teeth [15, 16-17]. Some researchers examined the teeth's natural movement using power limbs attached with hooks positioned at precisely the same position as the centre of resistance [15, 16- 17]. In orthodontic study findings, the skeletal as well as dental reactions to mechanical stresses have been examined FEA [10, 11, 12-13]. Because of its intricate three-dimensional structure, this approach provides the best means of accurately modeling the teeth as well as periodontium [11, 12, 13-14]. Periodontal strains brought on by orthodontic treatment have been the subject of some research. FEA has frequently been used for assessing these stresses [10, 11, 12-13]. A popular scientific tool for determining strains as well as stresses in intricate structures, FEA is also used extensively in clinical studies. Splitting a complicated framework into smaller, simpler parts known as elements is the foundation of FEA concept [9, 10, 11-12]. In order to explain their physical reaction to an external force or movement, these elements are assigned parameters like the Young's modulus. Nodes connect all of the separate physical components to create a coherent mesh. Computer-solved numerical approaches can be used to calculate the stress-strain behaviour of each constituent under a load [13, 14-15]. Therefore, it is of interest to evaluate and compare the stress distribution in the periodontium during maxillary canine retraction between the force applied at the level of canine bracket and at the level of power arm using 3-D finite element analysis.

## Methods and Materials:

Thirteen male and twenty-two female patients who underwent orthodontic therapy involving retraction of both canine in maxillary arch were included in this study. Their average age was 19.21 ± 9.14 years. FEA approach was used to determine the CR of both canines of maxillary arch for each patient. Customized segmental T-loops specific for every patient were used to randomly assign one canine of maxillary arch to receive translation (TR) orthodontic movement and another canine of maxilla to controlled tilting (CT) orthodontic movement. The closing pressure exerted by the T-loop was about 150cN.

## Finite element study:

## Three-dimensional finite element model:

The maxillary arch and mandibular arch were captured on a CBCT scan. The CBCT was extracted from a continuing research investigation. The maxillary CBCT image was used to build a 3D geometric representation of the teeth of maxilla, maxillary arch and associated structures. The maxillary teeth's measurements were uniform. The CBCT picture has been submitted to software meant for editing and processing 3D image after being stored as DICOM format. The subdomains are referred to as the elements, while the tooth itself is referred to as a continuum or domain. Discretization is the term for this technique.

The components could come in different shapes and be 1-, 2- or 3-dimensional. The elements must only be connected at the crucial locations, known as nodes and must not overlap [2-3]. Meshing is the process of combining items at the node level and removing duplicate nodes. The ANSYS program was used to create the elements. The element's kind and form have an impact on how accurate the analysis is. Commercially known as SOLID 98, the element variety is a hexahedral polynomial element. These elements feature three degrees of freedom at each node, twenty nodes and quadratic response on deflection. There are 95,611 nodes and 22,784 elements in the mesh of the model of right canine of maxilla. The 3D finite model was created using Solid Works Software from "Dassault Systems Solid Works Corporation" (Waltham, MA 02451, USA). The same program was used to create separate 3D FEMs for the power arm, archwire, bracket and periodontium. It transpired that the PDL's thickness was consistent at 0.2 mm. a maxillary first-premolar extraction situation present bilaterally with 12 teeth built using a 0.017 x 0.025 stainless steel archwire placed in a 0.022 slot orthodontic bracket was the subject of the 3D FEM model.

E-chain place at canine bracket hook to maxillary first molar tube hooks. Two models were constructed with e-chain placed at power arm height 9 mm and 13 mm soldered to mesial wing to canine bracket and power arm soldered on molar tube hook distally at 5 mm and 9 mm respectively. Ansys Software from ANSYS Inc was used for Finite Element Analysis. 3D model was directly imported into the Ansys Software." The materials properties of each material were defined in the Ansys software. In order to emulate the model's constraints and stop it from moving freely, boundary limits were established. To prevent the tooth from moving freely, the nodes that are connected to the bone's outer layer are locked in every direction. By using the subsequent DOF (degree of freedom), the three-dimensional representation was constrained. The 3D FEM, symmetrical boundary circumstances and fixed supports were used, with fixed support placed at the model's base. The 3D finite model 0.17 x 0.025 SS archwire inserted in 0.022 slot bracket. Force application of 150 grams (1.47 N) through elastomeric chain place at canine bracket hook to maxillary first molar tube hook and elastomeric chain placed at power arm soldered to mesial wing to canine bracket and power arm soldered on molar tube hook distally. It was estimated that the coefficient of friction that existed between archwire and orthodontic bracket slots was 0.2. A three-dimensional finite element algorithm was used to conduct three-dimensional FEA under these circumstances.

## Statistical analysis:

All data was entered in MS excel sheet. SPSS version 23 was used for statistical analysis. Student t test was used statistical analysis. p≤0.05 was considered to be statistically significant.

## Results:

The stress areas in periodontium associated with force application at canine bracket hook level was approximately 35%, at power arm soldered to canine bracket at 9 mm level was approximately 60-65% and at power arm soldered to canine bracket at 13 mm level was 100%. The findings were significant statistically with maximum stress areas in periodontium was associated with force application at power arm soldered to canine bracket at 13 mm level and minimum stress area in periodontium was associated with force application at canine bracket hook level (Table 1). Maximum principle stress (tension side) observed in the periodontium after application of 150 grams of force at canine bracket hook level was 2.7 x 10-3 MPa. Similarly, at power arm attached to orthodontic canine bracket at 9mm level was 8.5 x 10-2MPa, at power arm attached to orthodontic canine bracket at 13 mm level was 6.4 x 10-1Mpa. The findings were significant statistically with maximum principle stress (tension side) observed in the periodontium at power arm soldered to canine bracket at 9mm level and minimum at canine bracket hook level (Table 2). Minimum principle stress (compression side) observed in the periodontium after application of 150 grams of force at canine bracket hook level was 2.6 x 10-2 MPa, at power arm attached to canine orthodontic bracket at 9mm level was 7.8 x 10-3MPa, at power arm attached to canine orthodontic bracket, at power arm attached to canine orthodontic bracket at 13 mm level was 5.7 x 10-2Mpa. The findings were significant statistically with minimum principle stress (compression side) observed in the periodontium at power arm at 9mm level and minimum at canine bracket hook level (Table 3). Von Misses stress observed in the periodontium after application of 150 grams of force at canine bracket hook level was 7.7 x 10-3 MPa, at power arm fixed to canine orthodontic bracket at 9mm level was 2.1 x 10-3 MPa, at power arm fixed to canine bracket at power arm soldered to canine orthodontic bracket at 13 mm level was 1.6 x 10-3 Mpa. The findings were significant statistically with Von Misses stress observed in the periodontium at power arm fixed to canine orthodontic bracket at 9 mm level and minimum at canine bracket hook level (Table 4).

## Discussion:

Alveolar bone resorption and deposition are the results of a biological response to pressure in the periodontal ligament that causes tooth movements [14, 15-16]. Furthermore, it was mentioned that when all of a tooth's points move in a parallel, linear pattern in accordance with the force path, the tooth is considered to be translated [17, 18- 19]. The authors pointed out that forces applied to a tooth can result in translation movement, rotation movement, or a mix of translation and rotation movement, depending on how the force's trajectory of action interacts with the tooth's CR' [20, 21-22]. Sliding orthodontic mechanics offer an opportunity to optimize the system of forces needed for physiological movement of teeth since it is simple to adjust the vertical location of retraction pressures by attaching varying lengths of power limbs to an arch wire [15, 16, 17- 18]. Using bonded power arms with hooks positioned at the same height as the centre of resistance, several researchers watched how the teeth moved naturally [21, 22-23]. The findings of our study are in agreement with other studies showing difference in stress distribution in the periodontium during Maxillary Canine retraction between the forces applied at the level of canine bracket and at the level of power arm using 3-D FEA [21, 22-23, 24- 25]. In line with our study, other literature also showed maximum stress areas in periodontium which was associated with force application at power arm soldered to canine bracket at 13 mm level and minimum stress area in periodontium was associated with force application at canine bracket hook level [22- 23]. In orthodontic research, the skeletal and dental reactions to mechanical forces have been examined using finite element analysis. Because of its intricate three-dimensional geometry, this approach provides the best means of accurately modelling the teeth and periodontium [19-20].

Periodontal strains brought on by orthodontic loading have been the subject of some research. To find these stresses, FEA has been utilized extensively. FEA is a widely used scientific method for figuring out stresses and strains in complex structures. It is also widely utilized in clinical research. The FEA approach is based on breaking down a complex framework into smaller, more manageable components called elements [20, 21, 22-23]. These elements are given parameters, such as the Young's modulus, to describe their physical response to an external force or movement. All of the disparate physical elements are joined by nodes to form a cohesive mesh. The stress-strain behaviour of each component under load can be determined using computer-solved numerical techniques. In orthodontics, the periodontium experiences stresses and strains when an ideal force is applied to a tooth [11, 12-13]. Therefore, a physician must understand the force mechanism and stress distribution in the periodontium [24- 25]. The findings of our study have similarity with the findings of other studies [20, 21, 22, 23, 24, 25]. It was observed that there was difference in Von Misses stress at the position of canine bracket and at the position of power arm using 3-D FEA [18, 19, 20, 21, 22- 23]. The authors noted that according to the interaction between the force's trajectory of action and the tooth's center of resistance (CR), forces imparted to a tooth can cause translation movement, rotation movement, or a combination of translation movement and rotation movement [10, 11- 12]. Many different bracket compositions and strategies have been created and updated since the initial introduction of the Andrews straight wire apparatus [17, 18- 19]. In sliding orthodontic mechanics, ideal retraction pressures can be provided anywhere on the vertical plane by a power limb that propels the teeth in an already programmed trajectory to achieve physiological movement, but in closing looping mechanics, triggered loop pressures would only function at the bracket position [19, 20-21]. Some researchers examined the teeth's natural movement using power limbs attached with hooks positioned at precisely the same position as the CR [11- 12, 13, 14].

## Conclusion:

Maximum stress areas in periodontium was associated with force application at power arm soldered to canine bracket at 13 mm level and minimum stress area in periodontium was associated with force application at canine bracket hook level. Maximum principle stress (tension side) and minimum principle stress (compression side) observed in the periodontium at power arm soldered to canine bracket at 9 mm level and minimum at canine bracket hook level.

## Figures and Tables

**Table 1:** Stress areas in the periodontium observed in the periodontium after application of 150 grams of force at different heights of the power arm

	**Force application at canine bracket hook level**	**Force application at power arm attached to orthodontic canine bracket at 9mm level**	**Force application at power arm attached to orthodontic canine bracket at 13 mm level**
Stress areas in the periodontium	Approx 35 %	Approx 60-65%	100%
t value	0.986		
df	5		
P value	0.001*		
*Statistically significant

**Table 2:** Maximum principle stress (tension side) observed in the periodontium after application of 150 grams of force at different heights of the power arm

	**Force application at canine bracket hook level**	**Force application at power arm attached to orthodontic canine bracket at 9mm level**	**Force application at power arm attached to orthodontic canine bracket at 13 mm level**
Maximum principle	2.7 X 10^-3^ MPa	8.5 X 10^-2^MPa	6.4 X 10^-1^Mpa
stress(tension side)			
t value	0.986		
df	5		
P value	0.001		

**Table 3:** Minimum principle stress (compression side) observed in the periodontium after application of 150 grams of force at different heights of the power arm

**Stress values in Peridontium (mpa)**	**Force application at canine bracket hook level**	**Force application at power arm attached to canine orthodontic bracket at 9mm level**	**Force application at power arm attached to canine orthodontic bracket at 13 mm level**
Minimum principle stress	2.6 X 10^-2^ MPa	7.8 X 10^-3^MPa	5.7 X 10^-2^Mpa
(compression side)			
t value	0.853		
df	8		
P value	0.001*		
*statistically significant

**Table 4:** Von Misses stress observed in the periodontium after application of 150 grams of force at different heights of the power arm

	**Force application at canine bracket hook level**	**Force application at power arm attached to canine orthodontic bracket at 9mm level**	**Force application at power arm attached to canine orthodontic bracket at 13 mm level**
Von Misses stress	7.7 X 10^-3^ Mpa	2.1 X 10^-3^ MPa	1.6 X 10^-3^ Mpa
t value	0.963		
df	5		
P value	0.001*		
*statistically significant
